# Roles of Human Endogenous Retrovirus-K-Encoded Np9 in Human Diseases: A Small Protein with Big Functions

**DOI:** 10.3390/v16040581

**Published:** 2024-04-10

**Authors:** Jiaojiao Fan, Zhiqiang Qin

**Affiliations:** Department of Pathology, Winthrop P. Rockefeller Cancer Institute, University of Arkansas for Medical Sciences, 4301 W Markham St., Little Rock, AR 72205, USA; jfan@uams.edu

**Keywords:** retrovirus, HERV-K, Np9, treatment, human diseases

## Abstract

Human Endogenous Retrovirus Sequences (HERVs) constitute up to 8% of the human genome, yet not all HERVs remain silent passengers within our genomes. Some HERVs, especially HERV type K (HERV-K), have been found to be frequently transactivated in a variety of inflammatory diseases and human cancers. Np9, a small protein translated from the HERV-K *env* reading frame, has been reported as an oncogenic protein and is present in a variety of tumors and transformed cells. The Np9 protein can crosstalk with many cellular factors and is involved in the pathogenicity of various diseases, including some oncogenic virus infections. In the current review, we summarize recent findings about Np9 clinical relevance/implications, its mediated cellular functions/mechanisms, and potential targeted therapies in development.

## 1. Introduction

Human endogenous retroviruses (HERVs) are a subgroup of retroviruses that integrated their sequences into the host genome after exogenous retrovirus infection millions of years ago, accounting for about 8–9% of the human genome [1]. Due to the accumulation of mutations, most HERVs are commonly inactive and unable to replicate. However, some HERVs still have open reading frames and retain the potential for protein expression [2]. HERVs are now classified into 22 independently acquired families based on the first-letter amino acid core of the tRNA of the primary binding site used by HERV to start reverse transcription [3]. Among these families, some, such as HERV-K, have been implicated in various human diseases through transactivation [4,5].

**Figure 1:**
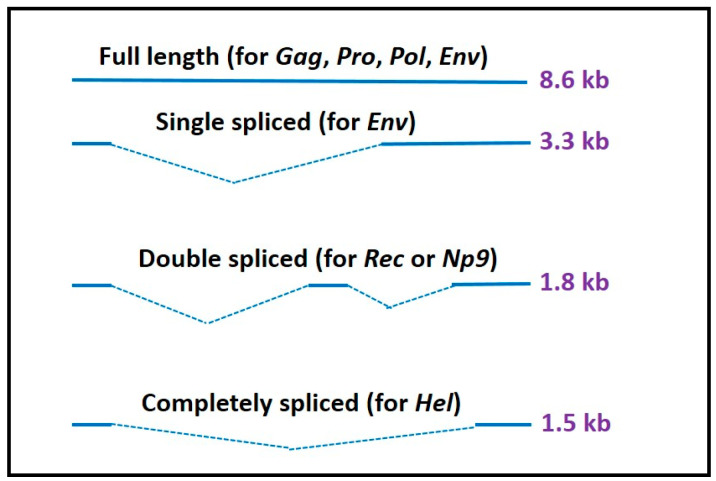
Different types of proviral transcripts of HERV-K (HML-2). The HERV-K (HML-2) usually expresses a full-length transcript (8.6 kb) and encodes the Gag, Pro, Pol and Env polyproteins. *Env* gene transcripts two singly spliced products, a 3.3-kb product to encode Env polyprotein, and a 1.5 kb product of unknown function known as the *hel* transcript, and a doubly spliced product (1.8 kb) to encode either the Rec or Np9 accessory proteins depending on the presence or absence of a 292-bp deletion at the *pol*/*env* boundary.

Similar to integrated retroviruses, a complete sequence of HERVs is mainly composed of *gag*, *pro*, *pol*, and *env* regions sandwiched between two long terminal repeats (LTRs). LTRs contain main promoters, enhancers, and transactivation regions for HERV transcription, thus regulating activation and expression of HERV genes [6]. Among HERV members, HERV-K (HML-2) has two major types of proviruses (type I and II), distinguished by the presence (type I) or absence (type II) of a 292-bp deletion at the *pol*/*env* boundary encoding two variant proteins, Np9 and Rec, respectively [7,8]. Unlike the type II provirus, which produces the regulatory protein Rec, the type I provirus produces the Np9 protein through a doubly spliced transcript in the *pol*/*env* boundary region (Figure 1). HERV-K (HML-2) also expresses a 1.5-kb transcript with an unknown function referred to as the *hel* transcript [7].

Although the Np9 protein is a small protein (only 9 kDa) translated from the HERV-K *env* reading frame, it has been found to be a multifunctional regulator that controls many downstream signaling pathways in host cells, and is involved in a variety of human diseases. In the current review, we summarize recent findings about Np9 clinical relevance/implications, its mediated cellular functions/mechanisms, and targeted therapies in development.

## 2. Clinical Relevance of Np9 in Human Diseases

In a recent study, Tavakolian et al. revealed a high transcription level of Np9 (but not Rec) in breast cancer tissues, including some late-stage patients, by RT-qPCR. They also found that the level of Np9 was associated with precancerous lesions of breast cancer [9]. These results suggest a plausible correlation between the mRNA level of Np9 and the progression of breast cancer, which may serve as a promising biomarker for diagnosing breast cancer. To study the possible relationship between HERV gene expression and chronic lymphocytic leukemia (CLL), Fischer et al. collected peripheral blood samples from patients with CLL and healthy donors. They found that the transcriptional activity of Np9 was greater in patients with CLL than in healthy donors. In contrast, gag expression was not significantly increased in patients [10]. Other studies also reported high expression of Np9 in blood samples from patients with digestive tract cancers such as gastric, colon, and pancreatic cancer, proposing the diagnostic value of Np9 in the early stage of gastrointestinal cancers [11,12]. Additionally, several studies reported Np9 expression in some melanoma tissues and/or melanoma cell lines, indicating that Np9 has a potential role in the development of melanoma [13,14]. Kaposi’s Sarcoma-associated Herpesvirus (KSHV, also named Human Herpesvirus 8, HHV-8) represents a principal causative agent of cancers arising in patients with compromised immune systems, including Kaposi’s Sarcoma (KS) and Primary Effusion Lymphoma (PEL) [15,16]. Our group recently found strong expression of Np9 within AIDS-KS tumor tissues (mostly in tumor cells, with low expressional levels in paired adjacent normal tissues) from a cohort of HIV+ patients without any treatment [17]. Using peripheral blood mononuclear cells (PBMCs) collected from a cohort of HIV+ patients, we found a higher level of HERV-K *env* transcripts in the KSHV+ group than in the KSHV− group [17]. Since there are no significant differences in HIV viral loads and CD4 counts between these two groups, we reason that KSHV infection is likely one of the driving forces for HERV-K transactivation in these patients. However, the level of Np9 was not detected in this study yet, which requires further investigation.

## 3. The Molecular Mechanisms of Np9-Mediated Pathogenesis and Oncogenesis

The Np9 protein can promote tumor cell survival or proliferation indirectly by activating other oncogenes and related signaling pathways. The published literature showed that the Np9 protein functionally interacted with the promyelocytic leukemia zinc finger (PLZF) tumor suppressor, a transcriptional repressor and chromatin remodeler implicated in cancer. Co-expression of Np9 with PLZF abrogated the transcriptional repression of the c-Myc gene promoter by PLZF and resulted in c-Myc overexpression, altered expression of c-Myc regulated genes, and ultimately promoted lymphoma cell proliferation and survival [18]. Furthermore, Chen et al. reported that Np9 not only increased leukemia cell growth by the activation of multiple oncogenic pathways, such as the Wnt/β-catenin and MAPK-ERK1/2 pathways, but also disrupted the Numb/Notch1 pathway, which participates in cellular metabolism and immunoregulation, by destabilizing an E3 ubiquitin ligase, LNX1 [19,20]. These data together indicate Np9 protein as a potential therapeutic target for the treatment of leukemia. Np9 can also directly bind to another ubiquitin ligase and p53 negative regulator, MDM2, to inhibit its ubiquitin ligase activity toward p53 and support the transactivation of genes by p53 [21]. Chan et al. reported that the depletion of Np9 increased the sensitivity of NCCIT teratocarcinoma cells to bleomycin and cisplatin [22]. Np9 was found to be essential for the migration of NCCIT teratocarcinoma cells using a wound closure assay: reduced expression of Np9 resulted in cells migrating into the wound at a slower rate.

**Figure 2:**
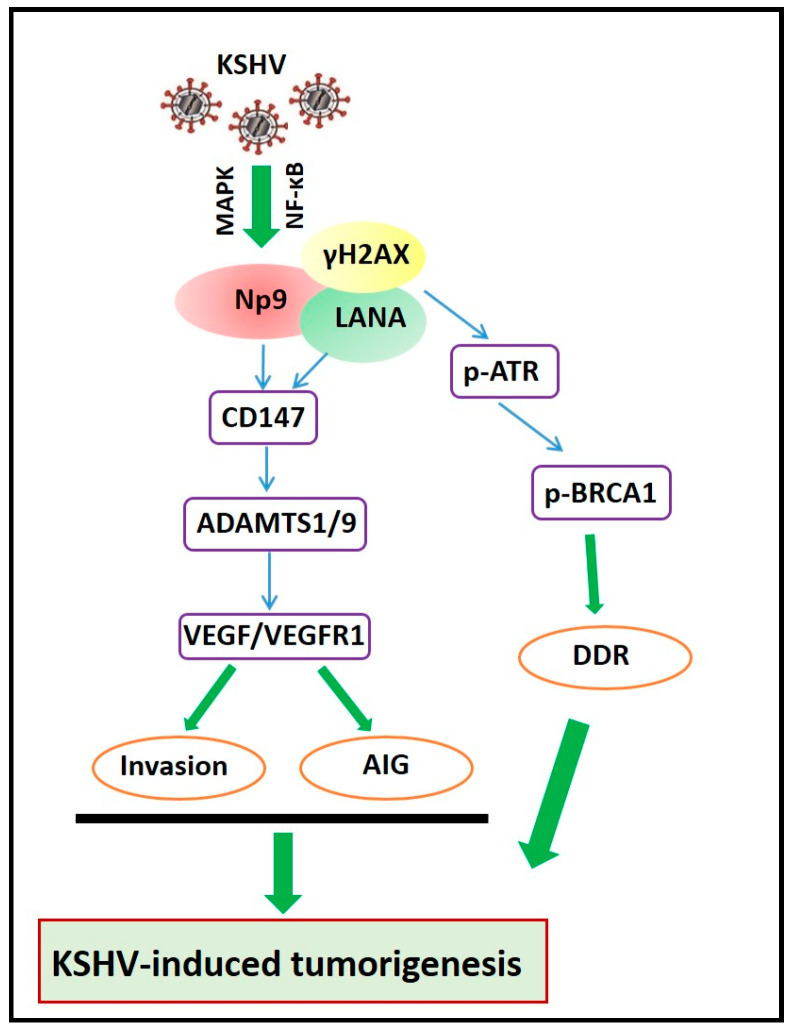
Schematic diagram of potential mechanisms for activation of Np9 protein promoting KSHV-induced tumorigenesis. AIG: anchorage-independent growth; DDR: DNA damage response.

Np9 also plays a role in the pathogenesis of some oncogenic viruses. Our previous study reported that KSHV de novo infection induced a gradient increase of Np9 transcripts from human umbilical vein endothelial cells (HUVECs) [17]. Notably, Np9 protein was only expressed in KSHV-infected primary cells, while none was seen in the uninfected cells. We also found increased primary endothelial cell invasion and anchorage-independent growth abilities by ectopic expression of Np9, potentially through the upregulation of one multifunctional glycoprotein, CD147 (also named as Emmprin), and its downstream signaling molecules including ADAMTS1 and ADAMTS9, two specific disintegrin and metalloproteinases [23]. Our recent study found that ectopic expression of Np9 protein was able to induce DNA damage response from host cells, especially through the upregulation of γH2AX [24]. Additionally, we found that Np9 was exclusively expressed in the nucleus, having a direct interaction with latency-associated nuclear antigen (LANA), one of the major latent proteins encoded by KSHV. Using a KS-like xenograft model [25], we found that silencing of Np9 by RNAi significantly repressed KSHV-induced tumorigenesis in nude mice [17]. Mice injected with Np9 stably knock-down TIVE-LTC (a KSHV long-term-infected telomerase-immortalized human umbilical vein endothelial cell line) formed much smaller tumors compared to mice injected with control shRNA cells at 30 days post-infection. Direct knockdown of Np9 effectively reduced LANA expression in KSHV+ tumor cells in vivo. These data together strongly support the important role of the activation of Np9 expression as a cofactor for KSHV-induced tumorigenesis (Figure 2).

Intriguingly, Gross et al. reported a strong upregulation of Np9 protein in Epstein–Barr virus (EBV)-transformed lymphocytes [26]. They found that Np9 could bind to the EBV-encoded nuclear antigen 2 (EBNA2) and negatively affected the EBNA2-mediated activation of the viral C- and latent membrane protein 2A (LMP2A) promoters. The downregulation of EBNA2 activity by Np9 might represent a cellular defense mechanism against viral infection. Therefore, Np9 protein may have diverse functions in different oncogenic virus infections.

## 4. Development of Np9-Targeted Therapies

Currently, there are few reports about Np9 inhibitors or the development of Np9-targeted therapies, as most studies focus on reducing or eliminating Np9 expression using RNAi or other gene editing technologies. Triptolide, a diterpenoid triepoxide, is purified from the roots of the Chinese herb *Tripterygium wilfordii* [27]. Triptolide displays a broad-spectrum bioactivity profile; for example, it has been reported to inhibit proliferation and induce apoptosis in a variety of tumor cell lines in vitro, including cholangiocarcinoma, melanoma, breast, gastric cancer, and non-small-cell lung carcinoma [28]. Chen et al. reported that Triptolide treatment induced apoptosis of human acute T lymphocytic leukemia (TLL) Jurkat cells through the inhibition of Np9 and downstream signaling molecules, c-Myc, β-catenin, ERK, Akt, and Notch1 activities [29]. Interestingly, another study reported that Triptolide treatment reduced LANA expression and displayed effective anti-PEL activities in vitro and in vivo [30], which is probably through the inhibition of Np9 expression and functions. However, the detailed interaction between Triptolide and Np9 protein still requires further investigation, and it remains unclear whether Triptolide treatment is also effective against KS.

## 5. Conclusions

In recent years, the reactivation of HERV-K (especially the activation of Np9 protein expression) has been increasingly linked to a range of human diseases. As the major oncoprotein of HERV-K, Np9 has been found to crosstalk with many cellular factors (e.g., oncogenes or tumor suppressors) and be involved in the pathogenicity of various diseases, although the underlying mechanisms are not fully understood. One of the major challenges is the lack of specific inhibitors targeting Np9 or the development of Np9-targeted therapies. For instance, there is an expectation to develop promising strategies to identify novel small-molecule Np9 inhibitors through high-throughput screening. Other recently developed technologies such as proteolysis-targeting chimeras (PROTACs), which are heterobifunctional small molecules composed of a protein of interest (POI) ligand as a warhead, a linker, and an E3 ligase ligand [31], may be considered. Additionally, it may be considered to combine Np9-targeted therapy with other therapies, including immunotherapy, since the reactivation of HERV-K has been found to have immunoregulatory functions in various human diseases [32,33]. It will be also interesting to explore and compare the expression and functions of Np9 with Rec or Hel in future studies.

## Data Availability

All the data shown in this paper are available from the corresponding authors upon reasonable request.
